# Sex Differences in Plaque Composition and Morphology Among Symptomatic Patients With Mild-to-Moderate Carotid Artery Stenosis

**DOI:** 10.1161/STROKEAHA.121.036564

**Published:** 2022-01-05

**Authors:** Dianne H.K. van Dam-Nolen, Nina C.M. van Egmond, Kristine Dilba, Kelly Nies, Anja G. van der Kolk, Madieke I. Liem, M. Eline Kooi, Jeroen Hendrikse, Paul J. Nederkoorn, Peter J. Koudstaal, Aad van der Lugt, Daniel Bos

**Affiliations:** Department of Radiology and Nuclear Medicine (D.H.K.v.D.-N., N.C.M.v.E., K.D., A.v.d.L., D.B.), Erasmus University Medical Center Rotterdam, Rotterdam, the Netherlands.; Department of Neurology (D.H.K.v.D.-N., P.J.K.), Erasmus University Medical Center Rotterdam, Rotterdam, the Netherlands.; Department of Epidemiology (D.B.), Erasmus University Medical Center Rotterdam, Rotterdam, the Netherlands.; Department of Radiology and Nuclear Medicine, CARIM School for Cardiovascular Diseases, Maastricht University Medical Center, the Netherlands (K.N., E.K.).; Department of Radiology, University Medical Center Utrecht, the Netherlands (A.G.v.d.K., J.H.).; Department of Radiology, Radboud University Medical Center, Nijmegen, the Netherlands (A.G.v.d.K.).; Department of Neurology, Academic Medical Center, Amsterdam, the Netherlands (M.I.L., P.J.N.).

**Keywords:** atherosclerosis, carotid stenosis, computed tomography angiography, hemorrhage, lipid, magnetic resonance imaging, sex characteristics

## Abstract

**Background and Purpose:**

Incidence of ischemic stroke differs between men and women, with substantially higher rates in men. The underlying mechanism of this difference remains poorly understood but may be because of differences in carotid atherosclerosis. Using an in-depth imaging-based approach, we investigated differences between carotid plaque composition and morphology in male and female patients with stroke, taking into account differences in total plaque burden. Additionally, we investigated all possible within-artery combinations of plaque characteristics to explore differences between various plaque phenotypes.

**Methods:**

We included 156 men and 68 women from the PARISK (Plaque At Risk) study, a prospective cohort study of patients with recent ischemic cerebrovascular symptoms and <70% ipsilateral carotid stenosis. Plaque characteristics (intraplaque hemorrhage [IPH], lipid-rich necrotic core [LRNC], calcifications, thin-or-ruptured fibrous cap, ulcerations, total plaque volume) were assessed with magnetic resonance imaging and multidetector-row computed tomography angiography. We used multivariable logistic and linear regression analyses to assess sex differences in plaque characteristics.

**Results:**

We found significant difference in total plaque volume between men and women (β=22.9 mm^3^ [95% CI, 15.4–30.5]; mean volume in men 1399±425 mm^3^, in women 1011±242 mm^3^). Additionally, men were more likely to have IPH (odds ratio [OR]=2.8 [95% CI, 1.3–6.3]; IPH proportion in men 49%, in women 16%) and LRNC (OR=2.4 [95% CI, 1.2–4.7]; LRNC proportion in men 73%, in women 41%) even after adjustment for total plaque volume. We found no sex-specific differences in plaque volume-corrected volumes of IPH, LRNC, and calcifications. In terms of coexistence of plaque characteristics, we found that men had more often a plaque with coexistence of calcifications, LRNC, and IPH (OR=2.7 [95% CI, 1.2–7.0]), with coexistence of thin-or-ruptured fibrous cap/ulcerations, LRNC, and IPH (OR=2.4 [95% CI, 1.1–5.9]), and with coexistence of all plaque characteristics (OR=3.0 [95% CI, 1.2–8.6]).

**Conclusions:**

In symptomatic patients with mild-to-moderate carotid stenosis, men are more likely to have a high-risk carotid plaque with IPH and LRNC than women, regardless of total plaque burden. Men also have more often a plaque with multiple vulnerable plaque components, which could comprise an even higher stroke risk.

**Registration:**

URL: https://www.clinicaltrials.gov; Unique identifier: NCT01208025.

## Abstract 2

Supplemental Digital Content is available in the text.

Ischemic stroke is leading cause of long-term disability and major cause of death worldwide. In terms of stroke occurrence, there are substantial differences between men and women, which remain poorly understood. Men, for example, have a 33% higher lifetime risk of stroke than women, but if women suffer from a stroke, its course is often more severe with a higher chance of permanent disability.^1–3^ An important explanation for these dissimilarities may be found in sex-specific differences in one of the major causes of stroke, namely carotid atherosclerosis.^4^

**See related article, p 379**

In brief, carotid atherosclerosis may result in a stroke through rupture of a carotid atherosclerotic plaque, which leads to embolization of plaque material and thrombus into distal arteries. Those plaques, which are more prone to rupture and are associated with higher risk for ischemic stroke, are known as vulnerable plaques. Important characteristics of these vulnerable plaques are the presence of intraplaque hemorrhage (IPH), a lipid-rich necrotic core (LRNC), a thin-or-ruptured fibrous cap (TRFC), and plaque ulceration.^5–7^ These plaque features can be visualized and quantified by multidetector-row computed tomographic angiography (MDCTA) and magnetic resonance imaging (MRI).^8^

Histopathologic ex vivo studies of carotid endarterectomy specimens have shown that women have a less vulnerable plaque phenotype characterized by decreased inflammatory mediators, lipids, and hemorrhage and increased fibrous tissue.^9^ Several imaging studies have confirmed these results and have reported that IPH, LRNC, and TRFC are more common in men than in women.^10–12^

However, sex differences in plaque morphology, characterized by features such as plaque ulceration, have not been well investigated yet. Moreover, previous studies have made no adjustments for the total plaque burden, although this plaque feature is strongly associated with plaque vulnerability and differs also significantly between men and women. Hence, the remaining question is whether sex differences in plaque composition cannot simply be explained by the fact that men have a larger plaque than women.^10^ This study aims to provide an extensive analysis on the differences between men and women in carotid plaque morphology and composition and, furthermore, to investigate whether sex differences in total plaque burden may explain sex differences in plaque characteristics.

## Methods

### Study Population

Patients were participants from the PARISK study (Plaque At RISK). The PARISK study is a prospective multicenter cohort study with the aim to improve the identification of patients with a high recurrent stroke risk with a mild-to-moderate carotid artery stenosis (<70%), using noninvasive imaging techniques. All included patients had a recent (<3 months) transient ischemic attack, including amaurosis fugax, or minor stroke, and a stenosis of <70% in the ipsilateral carotid artery. Institutional Review Board approval was obtained and all patients gave written informed consent. The study design protocol has been previously described in detail.^13^ The reporting of this study conforms to the STROBE statement (Supplemental Material).

**Figure 1.**
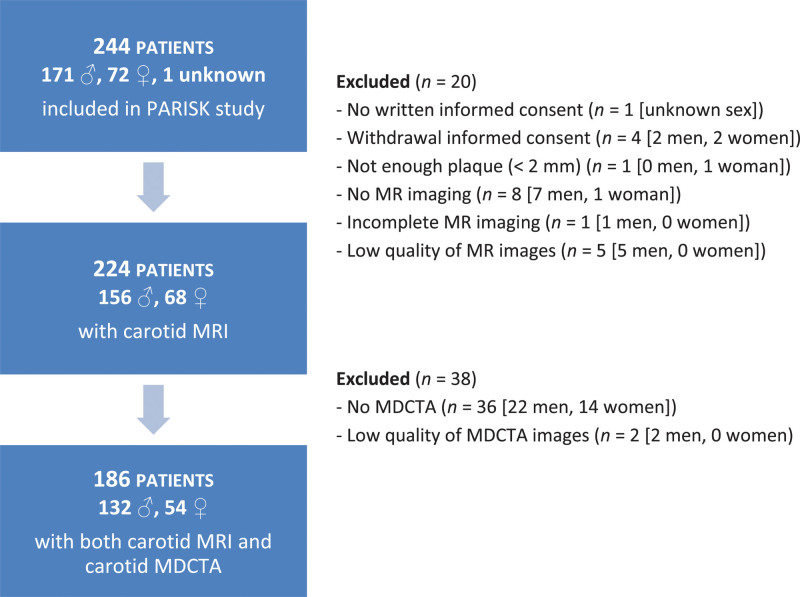
**Flowchart of patients included in the analyses.** One hundred eighty-six patients have both magnetic resonance imaging (MRI) and multidetector-row computed tomographic angiography (MDCTA) of the carotid artery. PARISK indicates Plaque At RISK study.

A total of 244 patients were included in de PARISK study between September 2010 and December 2014. We selected patients with a baseline MRI scan of their carotid arteries (n=224), since most of the parameters and the total plaque burden were measured with MRI. Of these 224 patients, 186 also had a MDCTA scan. We included 224 patients in the main analyses (Figure 1). The data that support the findings of this study are available from the corresponding author upon reasonable request.

### Assessment of Carotid Atherosclerosis

Patients underwent a high-resolution contrast-enhanced MRI and contrast-enhanced MDCTA scan of their carotid arteries, using a previously described standardized protocol.^13^ Carotid MRI was performed on a 3-Tesla scanner using a phased-array carotid coil. All MR and MDCTA images were reviewed by trained readers blinded to clinical and other imaging data.

MDCTA images were evaluated with dedicated 3D analyses software (Syngo.via; Siemens, Erlangen, Germany). The stenosis in the symptomatic internal carotid artery and carotid bifurcation was measured according to the ECST (European Carotid Surgery Trial) criteria perpendicular to the central lumen line.^14^ In addition, we assessed the presence of plaque ulceration in the symptomatic carotid artery. The multiplanar reformatting application allowed analysis of the carotid arteries in oblique, coronal, and sagittal planes. We defined plaque ulceration as an extension of contrast material of >1 mm in the surrounding atherosclerotic plaque on at least 2 orthogonal slices.^15,16^ To quantify calcifications within 3 cm proximal and distal to the carotid bifurcation, we used a custom-made plug-in for the software ImageJ (National Institutes of Health, Bethesda, MD). A threshold of 600 Hounsfield units was used to differentiate calcifications from contrast material. Calcification volume was expressed in millimeter cube.^17^

MR images were evaluated with dedicated vessel wall imaging analysis software (VesselMass, Department of Radiology, Leiden University Medical Center, the Netherlands). The images were automatically registered by delineating the lumen and outer vessel wall of the symptomatic carotid artery. If needed, registration was manually corrected. IPH and LRNC components were manually segmented. IPH was considered to be part of the LRNC. Fifteen 2 mm transverse adjoining slices covering the entire ipsilateral plaque were annotated. Total plaque volume could be derived additionally from the annotations. We used total plaque volume, expressed in millimeter cube, as a proxy for the total plaque burden. In all patients in whom specific plaque components were present, we quantified the volume of these components. Volumes of plaque components were expressed in millimeter cube. Further details about the measurement of the plaque components has been described previously.^18,19^

The fibrous cap status of the symptomatic carotid plaque was assessed in plaques with identified LRNC. The fibrous cap status was considered as thin and/or ruptured when the fibrous cap on at least one slice was scored as being thin and/or ruptured. Otherwise, the fibrous cap was considered as thick and intact. A TRFC was defined as an absent or interrupted hyperdense signal area adjacent to the lumen (overlying the LRNC) on the postcontrast TSE T1-weighted images. The assessment of the fibrous cap status has been described in detail elsewhere.^20^

### Assessment of Covariables

Cardiovascular risk factors that were assessed included hypercholesterolemia, hypertension, diabetes, and smoking status. Also, body mass index, use of cardiovascular medication, and history of ischemic cardiovascular disease were recorded. Hypercholesterolemia was defined as total cholesterol of >5 mmol/L or the use of lipid-lowering drugs at the time of the cerebral ischemic event. Hypertension was defined as a systolic blood pressure ≥140 mm Hg or diastolic blood pressure ≥90 mm Hg on clinical examination or the use of antihypertensive medication. Diabetes was defined as a serum glucose level of >6.9 mmol/L, a 2-hour post load glucose level of >11.0 mmol/L, or the use of antidiabetic medication. Smoking status at the time of the event was dichotomized into current smoker or noncurrent smoker.

### Statistical Analysis

Continuous variables are presented as mean±SD or as median (interquartile range). Categorical variables are presented as number (%). To analyze differences in baseline clinical and plaque characteristics between both sexes Pearson χ^2^ tests, Student *t* tests, and Mann-Whitney *U* tests were used.

Next, we used multivariable logistic regression analyses for analyzing the association between sex and the presence of IPH, LRNC, TRFC, plaque ulceration, and calcifications. To analyze the association between sex and the volume of IPH, LRNC, and calcifications, we used multivariable linear regression analyses. IPH, LRNC, and calcifications volumes were log-transformed to deal with their skewed distribution. Linear and logistic regression analyses were adjusted for total plaque volume, because plaque volume is highly correlated with presence of vulnerable plaque components and is usually larger in men because of their larger artery size.^10,21,22^ We additionally explored the presence of interaction between sex and cardiovascular risk factors in the association with plaque characteristics by stratifying on age (median was 69 years), hypertension, hypercholesterolemia, diabetes, current smoking, and antithrombotic medication use.

In addition to investigation of single plaque characteristics, we also investigated all possible within-artery combinations of plaque characteristics, which enables us to explore differences between various vulnerable plaque phenotypes, giving insight in the nature of plaque vulnerability. Multivariable logistic regression analyses were performed to analyze the association between sex and these combinations, using the similar regression model as above. Correlations between the presence of plaque characteristics were assessed using Cramér’s V for the presence of plaque characteristics, and Pearson correlation coefficient for the quantitative plaque measures.^23^

Missing data among covariables were imputed using 5-fold multiple imputations. Outcome variables were not imputed. All statistical analyses were conducted using R statistical software (version 3.6.3; R Foundation for Statistical Computing, Vienna, Austria).

## Results

The clinical characteristics of the study population (n=224) are presented in Table 1. The mean age was 69±9 years and 156 patients (70%) were men. Men more often used statins and antithrombotic medication. Current smoking was more prevalent in women.

**Table 1.**
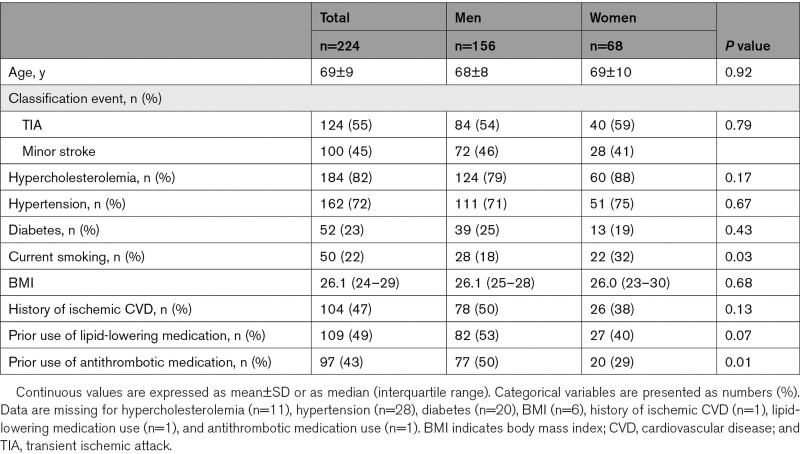
Baseline Clinical Characteristics

### Presence of Plaque Components

IPH, LRNC, and TRFC were significantly more prevalent in men than in women (IPH: 49% versus 16%, *P*<0.001; LRNC: 73% versus 41%, *P*<0.001; TRFC: 46% versus 28%, *P*=0.02). Ulcerations tended to be also more prevalent in men (32% versus 17%, *P*=0.06) (Table 2).

**Table 2.**
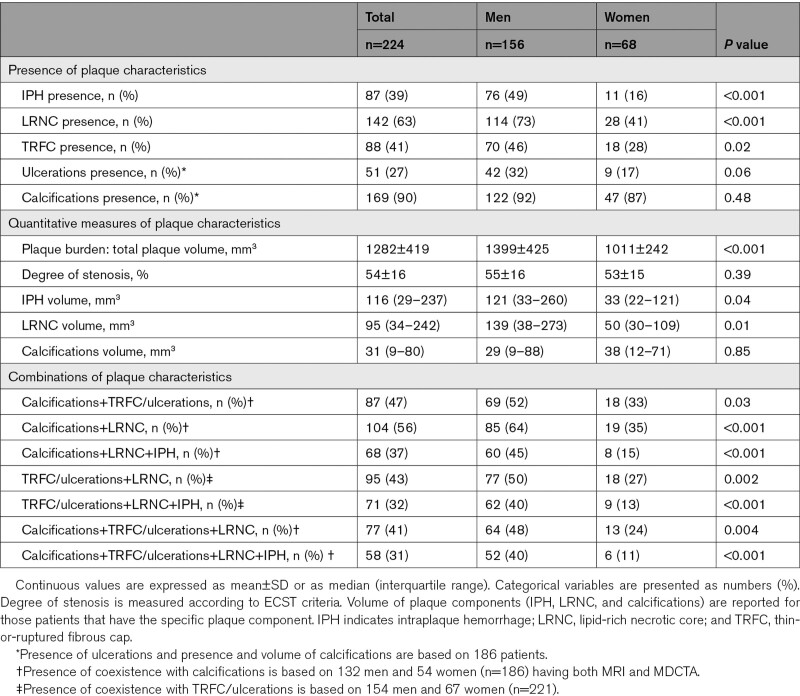
Baseline Plaque Characteristics

**Figure 2.**
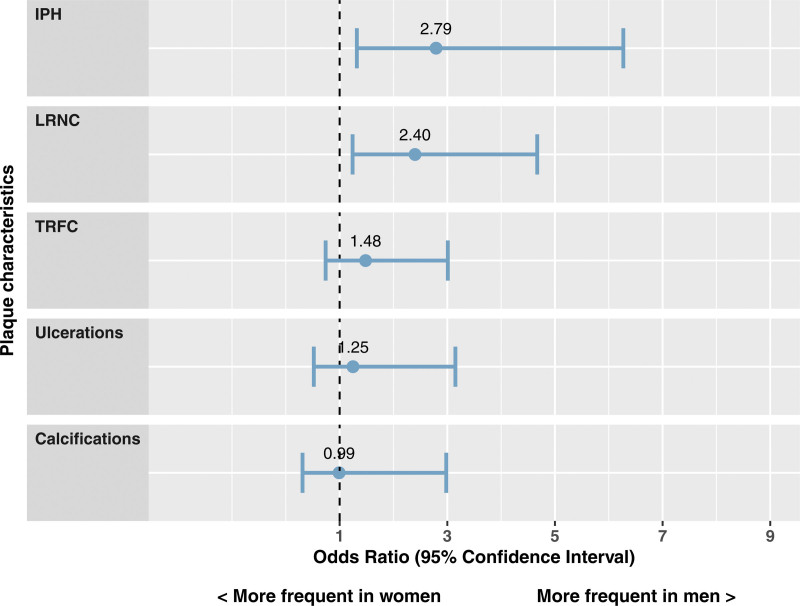
**Forest plot of association between sex and plaque characteristics.** Dependent variable is the presence of the plaque characteristics. Independent variable is sex with men as reference. Values are presented as odds ratio and 95% CIs. The model is adjusted for total plaque volume. IPH indicates intraplaque hemorrhage; LRNC, lipid-rich necrotic core; and TRFC, thin-or-ruptured fibrous cap.

We found significant difference in total plaque volume between men and women (β=22.9 [95% CI, 15.4–30.5]). Figure 2 shows the association of sex with the different plaque components. We found that men were more likely to have IPH and LRNC, even when adjusted for total plaque volume (odds ratio [OR]=2.8 [95% CI, 1.3–6.3] for IPH; OR=2.4 [95% CI, 1.2–4.7] for LRNC). TRFC and ulcerations were not significantly more prevalent in men when adjusted for total plaque volume. No sex differences were found in the prevalence of calcifications.

Table S1 shows the *P* for interaction between sex and cardiovascular risk factors in the association with plaque characteristics. Only the interaction term for sex with hypercholesterolemia in the association with calcifications was statistically significant (*P*=0.02).

### Volume of Plaque Components

**Figure 3.**
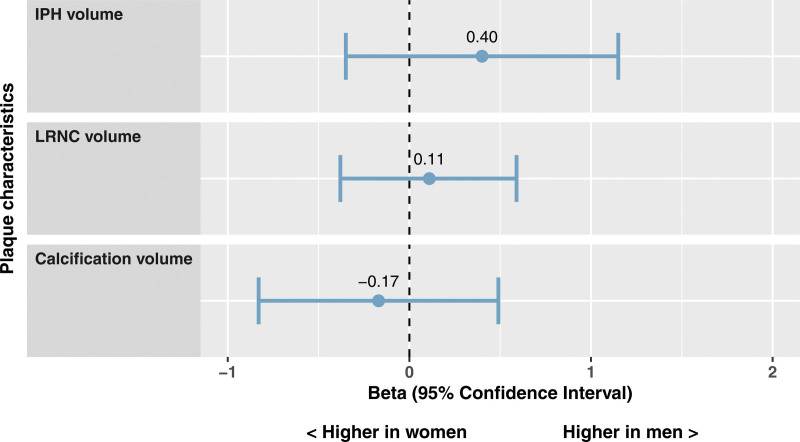
**Forest plot of association between sex and plaque component volumes.** Dependent variable is the volume of the plaque component. Independent variable is sex with men as reference. Values are presented as β and 95% CIs. Volumes of intraplaque hemorrhage (IPH), lipid-rich necrotic core (LRNC), and calcifications are Ln transformed. The model is adjusted for total plaque volume.

Figure 3 shows the multivariable regression analyses to evaluate the association between sex and plaque component volumes in patients having the plaque component of interest. No sex differences were observed in volumes of IPH, LRNC, and calcifications when adjusting for plaque burden.

### Correlation Between Plaque Characteristics

Presences of TRFC, IPH, and LRNC had strong correlations with each other (Cramér’s V was 0.6; Figure S1), both in men and women. In men, the correlations between presence of ulcerations and presence of IPH or TRFC had a Cramér’s V of 0.3, which are lower in women. The correlations between presence of calcifications and presence of ulcerations, LRNC, IPH, or TRFC ranged from 0.0 to 0.2.

Figure S2 shows the correlations between quantitative plaque measures. Both in men and in women we observed a very strong correlation between IPH and LRNC volume (Pearson’s correlation coefficient was 0.9, *P*<0.001). The correlations between IPH, LRNC, and calcification volume with total plaque volume ranged from 0.3 to 0.6. No correlation was seen for calcification volume with IPH or LRNC volume.

### Coexistence of Plaque Components

Table 2 shows the coexistence of several plaque features. We found that calcifications and LRNC most commonly coexisted (104 patients, 56%). Coexistence of TRFC/ulcerations with LRNC and coexistence of all plaque characteristics simultaneously were less frequent observed (respectively in 32% and 31% of the patients).

**Figure 4.**
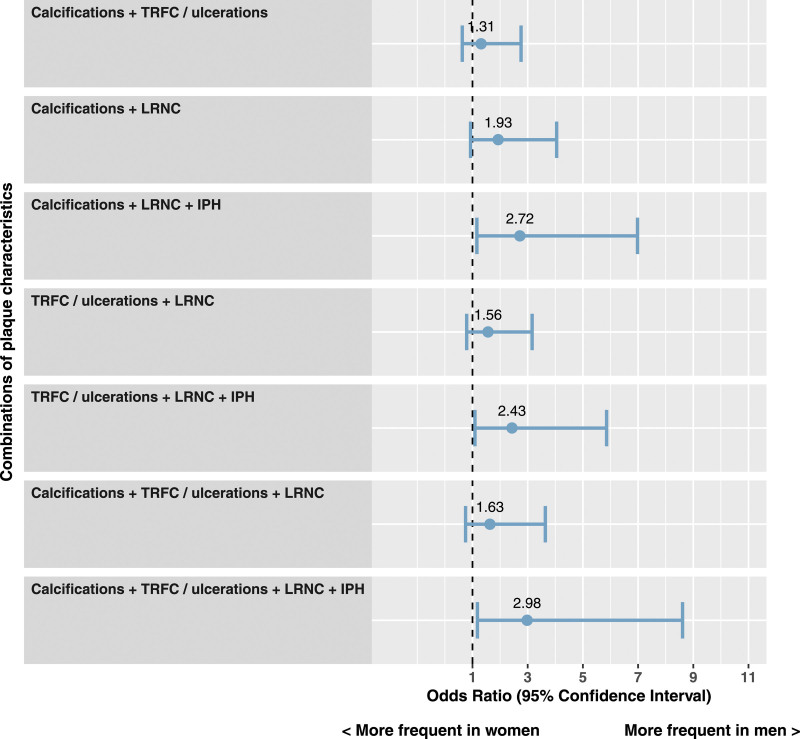
**Forest plot of association between sex and combinations of plaque characteristics.** Dependent variable is the presence of the combination of plaque characteristics. Independent variable is sex with men as reference. The model is adjusted for total plaque volume. IPH indicates intraplaque hemorrhage; LRNC, lipid-rich necrotic core; and TRFC, thin-or-ruptured fibrous cap.

Figure 4 shows the association between sex and several combinations of plaque characteristics. Men had more often coexistence of calcifications with LRNC and IPH (OR=2.7 [95% CI, 1.2–7.0]), of TRFC/ulcerations with LRNC and IPH (OR=2.4 [95% CI, 1.1–5.9]), and of all plaque characteristics, that is, calcifications with TRFC/ulcerations, LRNC and IPH (OR=3.0 [95% CI, 1.2–8.6]). The strongest difference between men and women was seen in this last combination of all vulnerable characteristics.

## Discussion

In this study, we showed sex differences in carotid plaque composition and morphology among symptomatic patients using high-resolution MRI and MDCTA. Men tended to have a larger plaque volume and a more vulnerable plaque composition. IPH, LRNC, and TRFC were more often seen in men than in women, and no sex differences were shown in calcification prevalence. Moreover, coexistence of vulnerable plaque characteristics was also more common in men.

Our results are in line with previous studies. Ota et al showed in asymptomatic individuals with <50% carotid stenosis that the presences of TRFC and IPH are higher in men than in women, and in asymptomatic patients with ≥50% carotid stenosis that TRFC and LRNC are more common in men.^11,12^ Furthermore, Van den Bouwhuijsen et al^10^ investigated subjects from the general population with atherosclerosis and found that men have a larger maximum wall thickness and a higher frequency of IPH and LRNC. All these studies also found no sex differences in the prevalence of calcifications. There are also some studies reporting no statistically significant associations between IPH, LRNC, TRFC, and sex.^24–26^ Nevertheless, often these studies show a tendency that vulnerable plaque characteristics are more common in men. We hypothesize that this could be caused by power issues. It would be of value to systematically analyze all literature on sex differences in carotid plaque characteristics to provide an overview and to reflect on research questions needing to be unraveled.

These findings have raised the question of whether sex differences in plaque component distribution are not just related to larger plaque burden in men. The larger plaque size in men could explain the higher prevalence of vulnerable plaque components in men, since these plaque characteristics are strongly associated with plaque burden.^21^ The majority of the previous studies have not adjusted their analyses for plaque burden. Our study shows that after adjusting for total plaque volume, the prevalence of IPH and LRNC remains still higher in men than in women, independent of plaque burden. Plaque burden plays a role in the relationship between sex and plaque components, but does not fully explain the sex differences.

We found a statistically significant interaction between sex and hypercholesterolemia in the association with calcifications. An explanation could lay in differences in lipid metabolism between men and women, which might be diminishing when using statins.^27^ Another explanation could be that our finding is just chance, since we tested multiple interaction terms.

To the best of our knowledge, our study is among the first studies that also investigated the distribution of sex differences in coexistence of various plaque components. Having a plaque with multiple vulnerable plaque components could comprise an even higher risk of rupture and ischemic stroke. Our finding of differences in the coexistence of various plaque characteristics supports the idea that men develop more vulnerable plaques: not only do they have a higher prevalence of either IPH, LRNC or TRFC, but also a higher prevalence of a carotid plaque with multiple vulnerable plaque characteristics simultaneously. It would be valuable to further investigate whether plaques with multiple vulnerable characteristics are indeed more prone to rupture than plaques with merely one of these characteristics.

Plaque characteristics showed strong to weak mutual correlations, the former suggesting that specific characteristics occur more frequently simultaneously, and the latter that there is also variety in type of atherosclerotic plaques. This also stresses the value of further investigating the coexistence of plaque characteristics.

No sex differences were found in the volumes of IPH, LRNC‚ and calcifications after adjustment for total plaque volume. Ota et al found in asymptomatic individuals with <50% stenosis that the relative volume of LRNC was higher in men than in women.^12^ However, these analyses were not adjusted. Our study confirms that LRNC volume differs between men and women, but that this difference disappears when correcting for plaque burden. Our results suggest that the differences in volume of IPH and LRNC are mainly explained by the plaque size and not by sex differences. Sex plays a role in developing a vulnerable plaque rather than in the size of the vulnerable plaque components. From a clinical point of view you could argue that the presence of plaque components is more important than the volume of these components. One reason for this is that it is easier to assess whether a feature is present (yes or no) than to measure the exact amount of that feature. Secondly, we do not know whether the volume of plaque components is an independent contributor to the risk of stroke. Most evidence from the literature points to IPH presence as an independent risk factor for new and recurrent stroke.^7^ Evidence is lacking for absolute or relative volumes of vulnerable plaque components as a predictor for stroke.

The finding that men more often have a vulnerable carotid plaque regardless of plaque size suggests that sex-specific management of stroke and patients with transient ischemic attack would be helpful to determine which patients would benefit from carotid revascularization. The higher occurrence of vulnerable plaques in men may be an explanation why carotid endarterectomy is reported as more effective in men than in women.^28^ The risk of plaque rupturing in men is probably higher than in women and, therefore, the risk reduction will also be higher. Tailoring cardiovascular risk management for men and women separately using the presence of vulnerable carotid plaque features is a promising strategy. This stresses the need for prospective studies on the causal relationship between these plaque features and stroke, taking into account sex-specific differences.

The strengths of this study includes the use of both MRI and MDCTA enabling us to assess multiple plaque characteristics, including ulcerations. Furthermore, this study also takes the role of plaque burden into account. Our findings suggest that having more vulnerable plaque components is not because of a larger plaque size. Another strength is the analyses of the coexistence of vulnerable plaque features, which gives us more insight into the nature of plaque vulnerability. Moreover, this study is a multicenter study which increases the generalizability of the findings.

Our study has some limitations. Firstly, the distribution of men and women included in our study is not equal. We think this could be because of various reasons, for example by differences in stroke incidence, by differences in willingness to participate, or by survivorship bias. For future studies, we recommend including a well-balanced male and female population to avoid power issues and to investigate a more representative cohort of patients. Another limitation is that we have no data about sex hormones and menopause status. It would be valuable for future research to evaluate the influence of these factors on the vulnerability of the plaque. Thirdly, this study has a cross-sectional design, and, therefore, we could make no causal inferences about the plaque characteristics and the risk of stroke. Future prospective studies may further elucidate sex-specific differences in the cause-effect mechanism between plaque characteristics and stroke.

## Conclusions

In symptomatic patients with mild-to-moderate carotid stenosis, men are more likely to have a high-risk carotid plaque with IPH and LRNC than women, regardless of the total plaque burden. Men also have more often a plaque with multiple vulnerable plaque components which could comprise an even higher stroke risk. These differences may help to explain sex differences in ischemic stroke and suggest that sex-specific tailoring of cardiovascular risk management using vulnerable plaque characteristics would be a promising strategy.

## Article Information

### Acknowledgments

Participating centers: Academic Medical Center, Amsterdam (P.J. Nederkoorn); Atrium Medisch Centrum, Heerlen (A.H.C.M.L. Schreuder); Erasmus MC, University Medical Center Rotterdam, Rotterdam (A. van der Lugt and P.J. Koudstaal); Flevoziekenhuis, Almere (M. Limburg); Kennemer Gasthuis, Haarlem (M. Weisfelt); Laurentius Ziekenhuis, Roermond (A.G. Korten); Maasstad Ziekenhuis, Rotterdam (R. Saxena); Maastricht University Medical Center (M.E. Kooi, R.J. van Oostenbrugge, W.H. Mess); Orbis Medisch Centrum, Sittard (N.P. van Orshoven); Sint Antonius Ziekenhuis, Nieuwegein (S.C. Tromp); Sint Franciscus Gasthuis, Rotterdam (S.L.M. Bakker); Slotervaartziekenhuis, Amsterdam (N.D. Kruyt); Tergooi Ziekenhuizen Hilversum/Blaricum (J.R. de Kruijk); University Medical Center Utrecht (J. Hendrikse, G.J. de Borst); Viecuri Medisch Centrum, Venlo (B.J. Meems); Vlietland Ziekenhuis, Schiedam (J.C.B. Verhey); IJsselland Ziekenhuis, Capelle aan den IJsel (A.D. Wijnhoud).

### Sources of Funding

This research was supported by the Dutch Heart Foundation and performed within the framework of the Center for Translational Molecular Medicine (www.ctmm.nl), project PARISK (Plaque At RISK; grant number 01C-202).

### Disclosures

None.

### Supplemental Material

STROBE Statement

Table S1

Figures S1–S2

## Supplementary Material


