# Supplementary material for: Sex Differences in Plaque Composition and Morphology Among Symptomatic Patients With Mild-to-Moderate Carotid Artery Stenosis
Source: Stroke. 2022 Jan 5;53(2):370–8. doi: 10.1161/STROKEAHA.121.036564 (PMC8785521; doi:10.1161/STROKEAHA.121.036564)
Supplement: Supplementary file 1 [file str-53-370-s001.pdf]

SUPPLEMENTAL MATERIAL

Sex Differences in Plaque Composition and Morphology among Symptomatic Patients with Mild-To-Moderate Carotid Artery Stenosis

Dianne H.K. van Dam-Nolen MD[a,b], Nina C.M. van Egmond BSc[a], Kristine Dilba MD[a], Kelly Nies MSc[c], Anja G. van der Kolk MD PhD[d,e], Madieke I. Liem MD[f], M. Eline Kooi PhD[c], Jeroen Hendrikse MD PhD[d], Paul J. Nederkoorn MD PhD[f], Peter J. Koudstaal MD PhD[b], Aad van der Lugt MD PhD[a], Daniel Bos MD PhD[a,g]

[a] Department of Radiology and Nuclear Medicine, Erasmus University Medical Center Rotterdam, Rotterdam, The Netherlands
[b] Department of Neurology, Erasmus University Medical Center Rotterdam, Rotterdam, The Netherlands
[c] Department of Radiology and Nuclear Medicine, CARIM School for Cardiovascular Diseases, Maastricht University Medical Center, Maastricht, The Netherlands
[d] Department of Radiology, University Medical Center Utrecht, Utrecht, The Netherlands
[e] Department of Radiology, Radboud University Medical Center, Nijmegen, Utrecht, The Netherlands
[f] Department of Neurology, Academic Medical Center, Amsterdam, The Netherlands
[g] Department of Epidemiology, Erasmus University Medical Center Rotterdam, Rotterdam, The Netherlands

Table of Contents

- STROBE Statement pp. 2-3
- Supplemental Table I p. 4
- Supplemental Figure I p. 5
- Supplemental Figure II p. 5

STROBE Statement – Checklist of items that should be included in reports of cross-sectional studies

	Item No	Recommendation	Page No
Title and abstract	1	(*a*) Indicate the study's design with a commonly used term in the title or the abstract	2
		(*b*) Provide in the abstract an informative and balanced summary of what was done and what was found	2-3
**Introduction**			
Background/rationale	2	Explain the scientific background and rationale for the investigation being reported	5-6
Objectives	3	State specific objectives, including any prespecified hypotheses	5-6
**Methods**			
Study design	4	Present key elements of study design early in the paper	6
Setting	5	Describe the setting, locations, and relevant dates, including periods of recruitment, exposure, follow-up, and data collection	6-8
Participants	6	(*a*) Give the eligibility criteria, and the sources and methods of selection of participants	6
Variables	7	Clearly define all outcomes, exposures, predictors, potential confounders, and effect modifiers. Give diagnostic criteria, if applicable	6-9
Data sources/ measurement	8*	For each variable of interest, give sources of data and details of methods of assessment (measurement). Describe comparability of assessment methods if there is more than one group	6-8
Bias	9	Describe any efforts to address potential sources of bias	8-9, 13-14
Study size	10	Explain how the study size was arrived at	6
Quantitative variables	11	Explain how quantitative variables were handled in the analyses. If applicable, describe which groupings were chosen and why	8-9
Statistical methods	12	(*a*) Describe all statistical methods, including those used to control for confounding	8-9
		(*b*) Describe any methods used to examine subgroups and interactions	8
		(*c*) Explain how missing data were addressed	9
		(*d*) If applicable, describe analytical methods taking account of sampling strategy	NA
		(*e*) Describe any sensitivity analyses	NA

**Results**			
Participants	13*	(a) Report numbers of individuals at each stage of study—eg numbers potentially eligible, examined for eligibility, confirmed eligible, included in the study, completing follow-up, and analysed	6, Fig. 1
		(b) Give reasons for non-participation at each stage	Fig. 1
		(c) Consider use of a flow diagram	Fig. 1
Descriptive data	14*	(a) Give characteristics of study participants (eg demographic, clinical, social) and information on exposures and potential confounders	9, Tab. 1, 2
		(b) Indicate number of participants with missing data for each variable of interest	Tab. 1, 2
Outcome data	15*	Report numbers of outcome events or summary measures	9-11 Tab. 2
Main results	16	(*a*) Give unadjusted estimates and, if applicable, confounder-adjusted estimates and their precision (eg, 95% confidence interval). Make clear which confounders were adjusted for and why they were included	9-11, Fig. 2-4
		(*b*) Report category boundaries when continuous variables were categorized	NA
		(*c*) If relevant, consider translating estimates of relative risk into absolute risk for a meaningful time period	NA
Other analyses	17	Report other analyses done—eg analyses of subgroups and interactions, and sensitivity analyses	9-11, Suppl. Material
**Discussion**			
Key results	18	Summarise key results with reference to study objectives	11-13
Limitations	19	Discuss limitations of the study, taking into account sources of potential bias or imprecision. Discuss both direction and magnitude of any potential bias	13-14
Interpretation	20	Give a cautious overall interpretation of results considering objectives, limitations, multiplicity of analyses, results from similar studies, and other relevant evidence	11-14
Generalisability	21	Discuss the generalisability (external validity) of the study results	11-14
**Other information**			
Funding	22	Give the source of funding and the role of the funders for the present study and, if applicable, for the original study on which the present article is based	15

*Give information separately for exposed and unexposed groups.

Note: An Explanation and Elaboration article discusses each checklist item and gives methodological background and published examples of transparent reporting. The STROBE checklist is best used in conjunction with this article (freely available on the Web sites of PLoS Medicine at http://www.plosmedicine.org/, Annals of Internal Medicine at http://www.annals.org/, and Epidemiology at http://www.epidem.com/). Information on the STROBE Initiative is available at www.strobe-statement.org.

Supplemental Table I. *P* interaction values of covariables with sex for the association with plaque characteristics

	IPH	LRNC	TRFC	Ulcerations	Calcifications
Age	0.45	0.23	0.09	0.81	0.72
Hypertension	0.37	0.61	0.78	0.84	0.99
Hypercholesterolemia	0.83	0.90	0.76	0.84	0.02
Diabetes Mellitus	0.65	0.39	0.13	0.99	0.99
Current smoking	0.33	0.74	0.91	0.77	0.76
Antithrombotic medication use	0.22	0.55	0.39	0.54	0.92

Shown are *P* values for interaction of age and major cardiovascular risk factors with sex on the association of sex with the presence of plaque characteristics. Interaction terms were considered significant when *P*-interaction < 0.05. IPH = intraplaque hemorrhage; LRNC = lipid-rich necrotic core; TRFC = thin-or-ruptured fibrous cap.

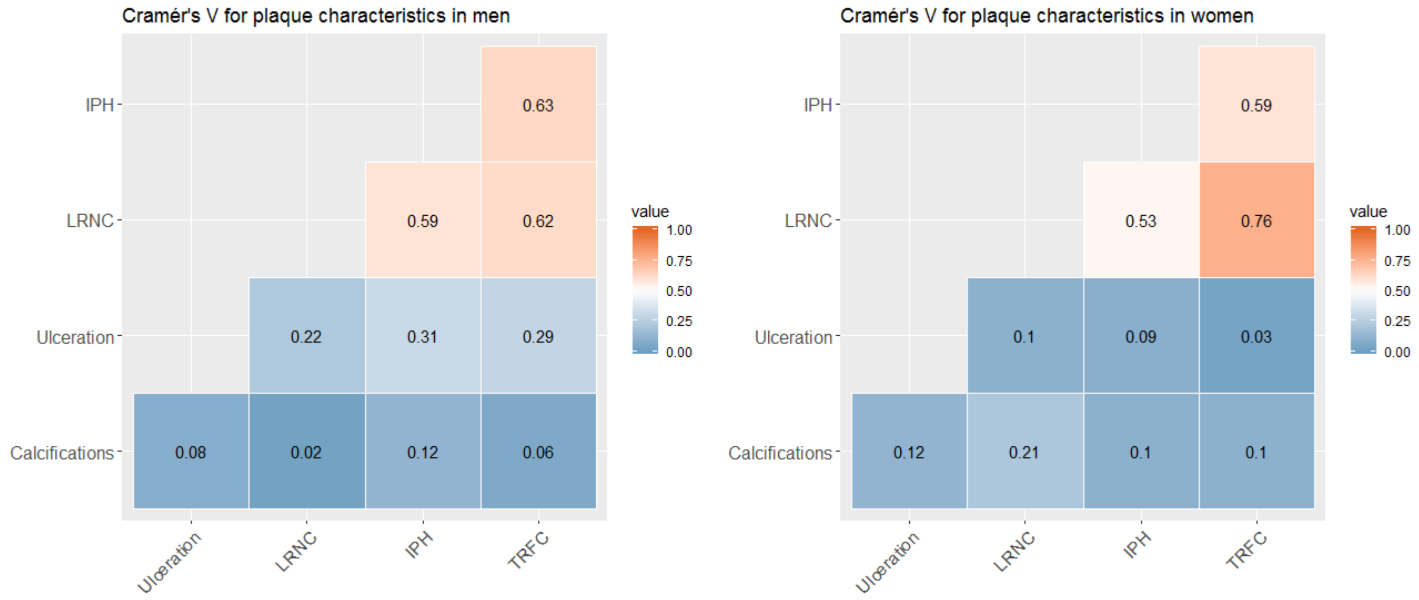

Supplemental Figure I. Plot of Cramér's V for plaque characteristics.

The plots show the correlation between the presence of different carotid plaque characteristics, stratified for men and women. IPH = intraplaque hemorrhage. LRNC = lipid-rich necrotic core, TRFC = thin-or-ruptured fibrous cap.

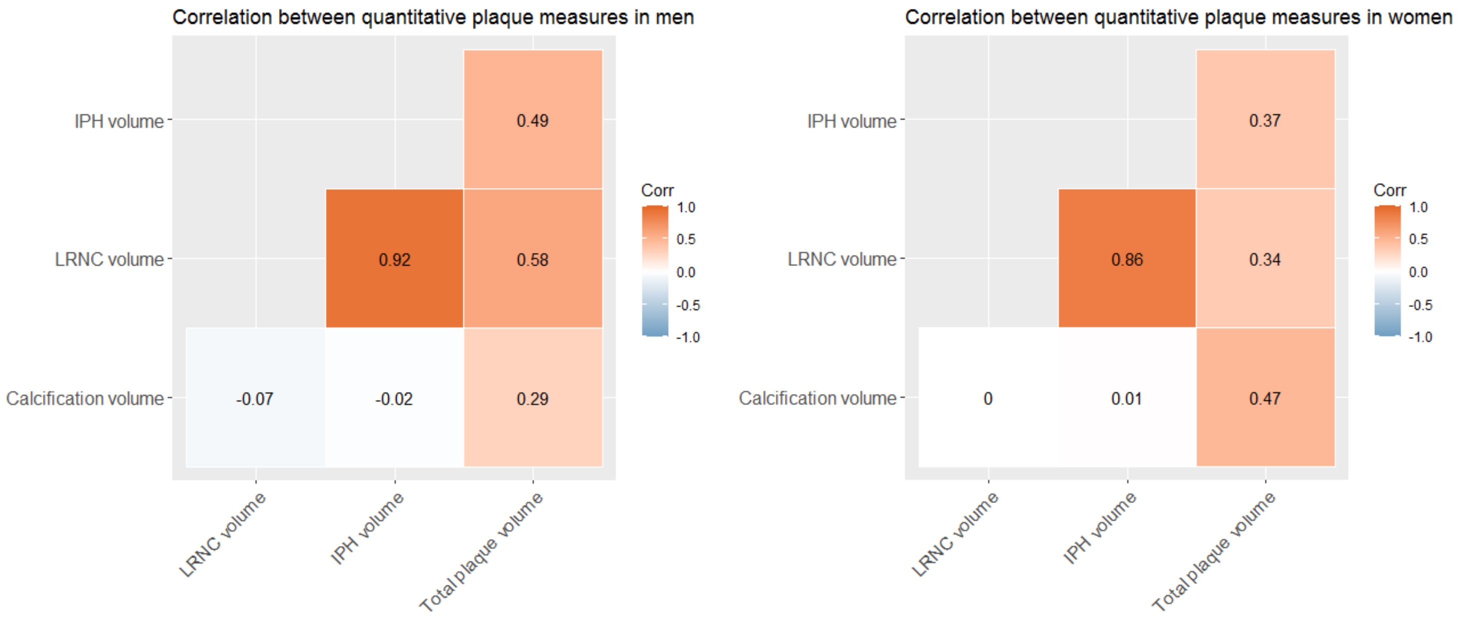

Supplemental Figure II. Correlation plots of quantitative plaque measures.

The plots show the Pearson's correlation coefficient between different quantitative plaque measures, stratified for men and women. IPH = intraplaque hemorrhage, LRNC = lipid-rich necrotic core.
