# Supplementary figures and images for: Sex Differences in Plaque Composition and Morphology Among Symptomatic Patients With Mild-to-Moderate Carotid Artery Stenosis
Source: Stroke. 2022 Jan 5;53(2):370–8. doi: 10.1161/STROKEAHA.121.036564 (PMC8785521; doi:10.1161/STROKEAHA.121.036564)

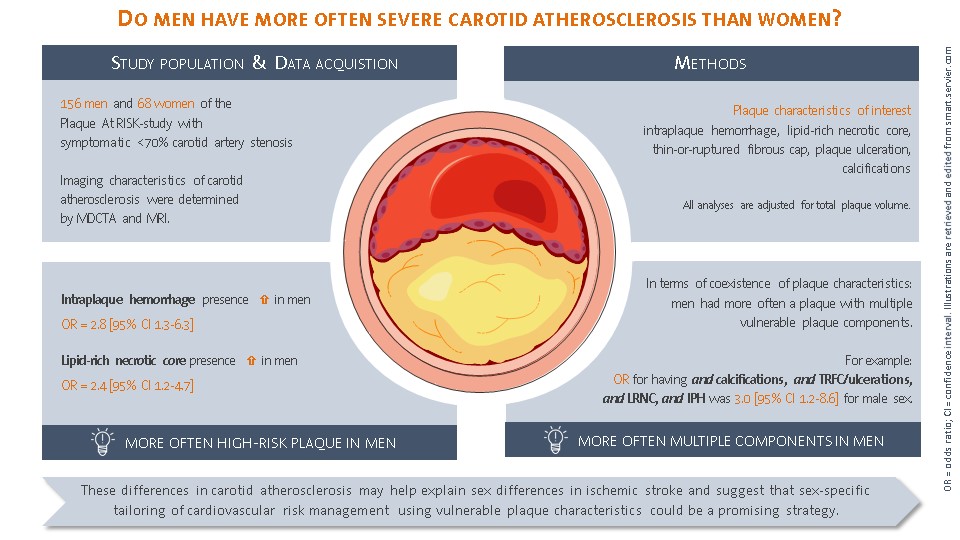

Supplement: Supplementary file 2 [file str-53-370-s002.jpg]
